# Pseudotumoral donovanosis mimicking squamous cell carcinoma in a patient with HIV infection

**DOI:** 10.1016/j.bjid.2026.105971

**Published:** 2026-09-26

**Authors:** Beatriz Cavalcante de Carvalho, Alex Panizza Jalkh, Alcidarta dos Reis Gadelha, Virginia Vilasboas Figueiras, Rima de Souza Raad

**Affiliations:** Fundação de Medicina Tropical Doutor Heitor Vieira Dourado, Manaus, AM, Brazil

**Keywords:** Donovanosis, HIV infections, Genital ulcer, Squamous cell carcinoma

## Abstract

Donovanosis is a chronic granulomatous sexually transmitted infection that may clinically mimic malignancy. We report a 64-year-old man with well-controlled HIV infection presenting with a chronic infiltrated vegetative scrotal ulcer showing progressive enlargement, exophytic growth, and hemorrhagic ulceration. Despite prolonged antimicrobial therapy with azithromycin and doxycycline, followed by adjunctive acyclovir because of suspected chronic herpes simplex coinfection, the lesion continued to progress, raising concern for squamous cell carcinoma. Initial histopathological and microbiological investigations, including cytology for Donovan bodies, were inconclusive. Repeat biopsy demonstrated pseudoepitheliomatous hyperplasia with a dense plasma cell-rich inflammatory infiltrate without cytological atypia, while periodic acid-Schiff staining identified intracellular Donovan bodies, confirming donovanosis. Doxycycline was reintroduced and maintained until complete healing after four months. This case highlights the diagnostic challenges of pseudotumoral donovanosis in patients living with HIV.

## Introduction

Donovanosis is a chronic granulomatous sexually transmitted infection caused by *Klebsiella granulomatis*.^1^ Although ulcerovegetative lesions represent the classical clinical presentation, chronic infiltrative lesions may occasionally resemble squamous cell carcinoma, particularly when they progressively enlarge despite prolonged courses of antimicrobial therapy, creating important diagnostic challenges, especially in patients living with HIV.^1^, ^2^, ^3^, ^4^, ^5^ We report an atypical pseudotumoral presentation of donovanosis with initially inconclusive investigations and continued progression despite prolonged courses of antimicrobial therapy.

## Case report

A 64-year-old man, originally from Haiti and residing in the Brazilian Amazon, with well-controlled HIV infection (undetectable viral load and CD4+ T-cell count of 527 cells/mm^3^), was referred for evaluation of a chronic vegetative scrotal ulcer. Physical examination revealed an infiltrated, exophytic lesion with a lobulated surface, progressive enlargement, and areas of hemorrhagic ulceration associated with easy bleeding (Figs. 1A–C).

**Fig. 1:**
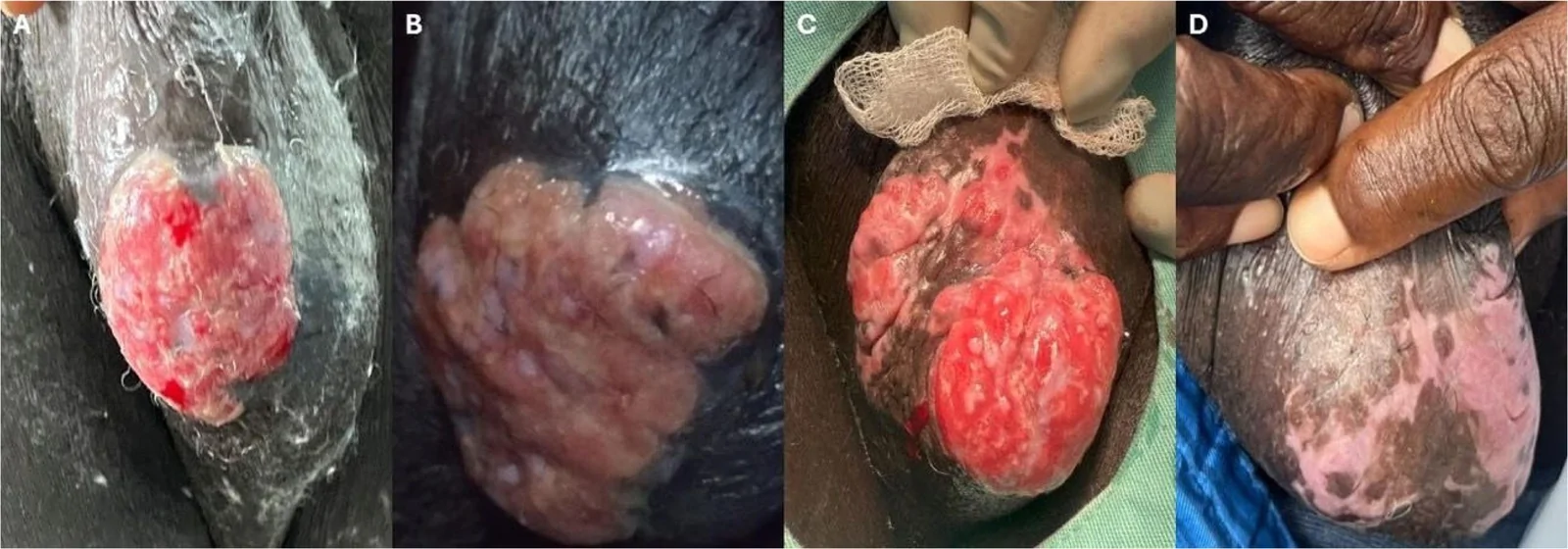
(A–C) Chronic infiltrated vegetative lesion with progressive exophytic growth and hemorrhagic ulceration, prompting clinical suspicion of squamous cell carcinoma. (D) Complete healing after prolonged doxycycline therapy.

Based on the clinical presentation, donovanosis was initially considered the most likely diagnosis, and a punch biopsy was performed. Histopathological examination demonstrated epidermal acanthosis with ulceration and a dense dermal inflammatory infiltrate composed predominantly of plasma cells, accompanied by lymphocytes and scattered eosinophils. Although the histopathological findings were compatible with donovanosis in the appropriate clinical context, the biopsy was not diagnostic, and the pathology report recommended clinicopathological correlation and further laboratory investigation before establishing a definitive diagnosis.

Initial cytological examination with Giemsa staining did not reveal Donovan bodies. Further investigations, including VDRL, direct examination and fungal cultures, bacterioscopy, and mycobacterial cultures, were negative. Adherence to treatment was confirmed; however, the lesion continued to progress despite prolonged courses of antimicrobial therapy, including azithromycin 1g weekly for four months and doxycycline 100 mg twice daily for two months, without clinical improvement. Considering possible concomitant chronic ulcerative herpes simplex infection, acyclovir 800 mg three times daily was added to antibiotic therapy for two months, again without improvement.

Despite confirmed adherence to prolonged antimicrobial therapy, the lesion continued to enlarge progressively, and neither the initial biopsy nor subsequent microbiological investigations established a definitive diagnosis. Given the persistent progression despite prolonged courses of antimicrobial therapy, infiltrative morphology with easy bleeding, and persistent diagnostic uncertainty, together with the need to exclude malignancy in a patient living with HIV, an incisional spindle biopsy was performed to exclude squamous cell carcinoma. Histopathology revealed epidermal acanthosis with ulceration but no cytological atypia (Fig. 1E). The dermis showed a dense inflammatory infiltrate rich in plasma cells, associated with neutrophilic collections (Figs. 2A–C). Periodic Acid-Schiff (PAS) staining demonstrated vacuolated histiocytes containing intracellular cocco-bacillary structures consistent with Donovan bodies (Fig. 3), confirming the diagnosis.

**Fig. 2:**
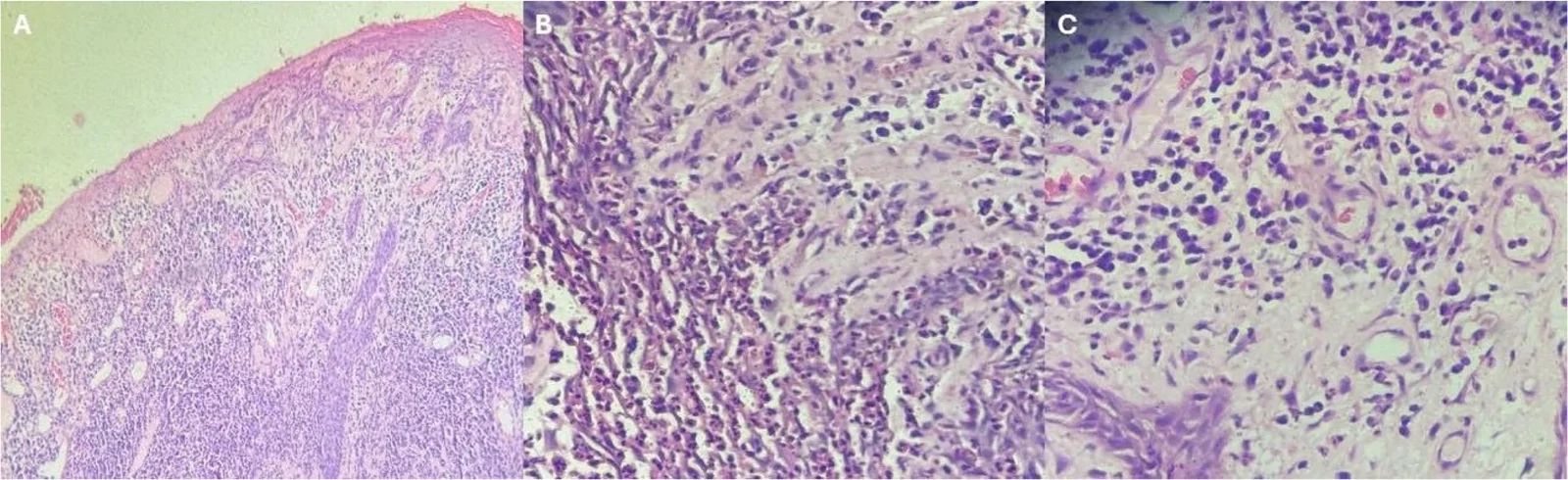
(A) Pseudoepitheliomatous hyperplasia with ulceration (H&E, ×10). (B) Neutrophilic collections within the dermal inflammatory infiltrate (H&E, ×400). (C) Dense plasma cell-rich inflammatory infiltrate (H&E, ×400).

**Fig. 3:**
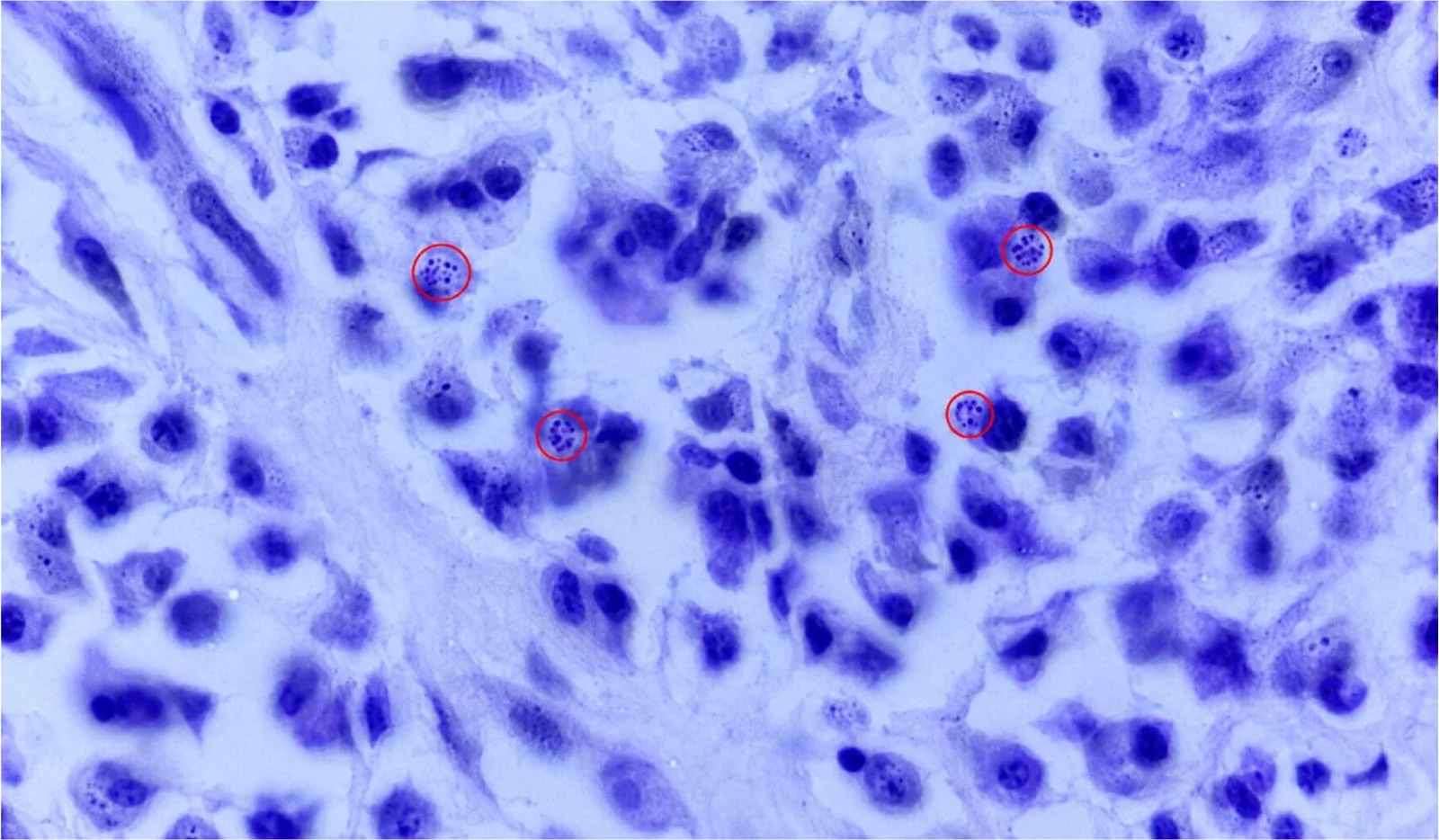
Periodic Acid-Schiff (PAS) stain demonstrating intracytoplasmic Donovan bodies (red circles) within macrophages, confirming the diagnosis of granuloma inguinale (original magnification ×1000, oil immersion).

Doxycycline (100 mg twice daily) was reintroduced and maintained until complete healing, achieved after four months (Fig. 1D).

## Discussion

Donovanosis is a chronic granulomatous infection that may present with exuberant vegetative lesions and clinically mimic malignant neoplasms, particularly squamous cell carcinoma.^1^^,^^3^ Although ulcerovegetative lesions are characteristic of donovanosis, the suspicion of squamous cell carcinoma in our patient was based not only on the clinical appearance but also on the infiltrative morphology, progressive enlargement, easy bleeding, continued progression despite prolonged courses of antimicrobial therapy, and persistent diagnostic uncertainty after repeated investigations. These findings justified repeat biopsy to exclude malignancy.^3^^,^^5^

The absence of cytological atypia and the identification of Donovan bodies on repeat biopsy were essential to exclude malignancy and establish the diagnosis. Similar diagnostic difficulties and occasional treatment resistance have been described, especially in immunocompromised patients.^1^, ^2^, ^3^, ^4^ HIV coinfection has been associated with reduced therapeutic response to azithromycin and prolonged treatment courses.^2^^,^^4^ Furthermore, people living with HIV are at increased risk of infection-related malignancies, reinforcing the need to exclude squamous cell carcinoma in chronic genital lesions that fail to respond to standard therapy.^6^

Although malignant transformation of chronic donovanosis is uncommon, it has been documented in longstanding lesions and should be considered when persistent progression despite prolonged courses of antimicrobial therapy raises concern for squamous cell carcinoma.^3^^,^^5^^,^^7^

This case emphasizes that donovanosis should remain in the differential diagnosis of chronic vegetative genital lesions, particularly when they are infiltrative, progressively enlarging, or continue to progress despite prolonged courses of antimicrobial therapy. In patients living with HIV, persistent diagnostic uncertainty after prolonged antimicrobial therapy should prompt repeat biopsy to exclude squamous cell carcinoma while avoiding unnecessary surgical intervention once the diagnosis is established.^1^^,^^3^^,^^8^

## Ethical approval

Ethical approval was not required for this case report according to institutional policies.

## Informed consent

Written informed consent was obtained from the patient for publication of this case report and accompanying images, including images of the genital region. All reasonable efforts have been made to ensure patient anonymity.

## Authors’ contributions

All authors contributed to conception, data collection, analysis, drafting, revision, and approval of the final manuscript.

## Funding

This research received no specific grant from any funding agency in the public, commercial, or not-for-profit sectors.

## Conflicts of interest

The authors declare no conflicts of interest.

## Data Availability

All data relevant to this case report are included within the article.
